# Multi-wavelength emission from a single InGaN/GaN nanorod analyzed by cathodoluminescence hyperspectral imaging

**DOI:** 10.1038/s41598-018-20142-5

**Published:** 2018-01-29

**Authors:** Gunnar Kusch, Michele Conroy, Haoning Li, Paul R. Edwards, Chao Zhao, Boon S. Ooi, Jon Pugh, Martin J. Cryan, Peter J. Parbrook, Robert W. Martin

**Affiliations:** 1 Department of Physics, SUPA, University of Strathclyde, Glasgow, G4 0NG United Kingdom; 2 Tyndall National Institute, University College Cork, Lee Maltings, Dyke Parade, Cork, Ireland; 3 School of Engineering, University College Cork, College Road, Cork, Ireland; 4 Pacific Northwest National Laboratory, Richland, WA United States of America; 5 Photonics Laboratory, King Abdullah University of Science and Technology (KAUST), Thuwal, 23955-6900 Saudi Arabia; 6 Department of Electrical and Electronic Engineering, University of Bristol, Bristol, BS8 1UB United Kingdom

## Abstract

Multiple luminescence peaks emitted by a single InGaN/GaN quantum-well(QW) nanorod, extending from the blue to the red, were analysed by a combination of electron microscope based imaging techniques. Utilizing the capability of cathodoluminescence hyperspectral imaging it was possible to investigate spatial variations in the luminescence properties on a nanoscale. The high optical quality of a single GaN nanorod was demonstrated, evidenced by a narrow band-edge peak and the absence of any luminescence associated with the yellow defect band. Additionally two spatially confined broad luminescence bands were observed, consisting of multiple peaks ranging from 395 nm to 480 nm and 490 nm to 650 nm. The lower energy band originates from broad *c*-plane QWs located at the apex of the nanorod and the higher energy band from the semipolar QWs on the pyramidal nanorod tip. Comparing the experimentally observed peak positions with peak positions obtained from plane wave modelling and 3D finite difference time domain(FDTD) modelling shows modulation of the nanorod luminescence by cavity modes. By studying the influence of these modes we demonstrate that this can be exploited as an additional parameter in engineering the emission profile of LEDs.

## Introduction

High quality InGaN/GaN LEDs are key to future lighting technologies, promising considerable reduction in worldwide power consumption. Nanorod LED arrays offer additional advantages over the commonly used planar LED structures. Firstly, nanorods offer a reduced density of threading dislocations, which can act as non-radiative recombination centers^1–3^, and thus achieve a higher internal quantum efficiency when compared to planar LEDs. Secondly, wurtzite nanorod structures grown in the *c*-direction offer easy access to semipolar and nonpolar facets on the pyramidal top of the nanorod or the sidewalls respectively. These facets offer strongly reduced electric fields, which are well known to cause the quantum confined Stark effect (QCSE), and reduce the recombination rate in *c*-plane planar LEDs^4^. Thirdly, nanorod structures can offer an increased active zone area compared to planar LEDs thereby increasing the output power per area.

Fourthly, nanorod structures can act as cavities in which optical modes form^5–7^. A number of publications have investigated these optical modes within different nanorod structures, including state of the art core-shell In_*x*_Ga_1−*x*_N nanorods, utilizing plane-wave approximations as well as advanced modelling tools such as finite dimension and time domain (FDTD) modelling^5,8,9^. Their findings show that strong coupling exists between light emitted from *m*-plane nonpolar QWs and the optical modes and that the resonant wavelengths can be tuned by the nanorod diameter.

This paper presents our investigation of light emitted from spatially separated active regions, with different polarities (*c*-plane and semipolar), of a single In_*x*_Ga_1−*x*_N/GaN nanorod from an ultra-dense nanorod array (space filling factor >96%), using cathodoluminescence (CL) hyperspectral imaging. Furthermore, it presents a detailed study of optical modes in the nanorod, utilizing plane-wave and finite dimension and time (FDTD) modelling, providing evidence for the light of the different active regions coupling into, and being modulated by, the same cavity mode.

## Results and Discussion

**Figure 1:**
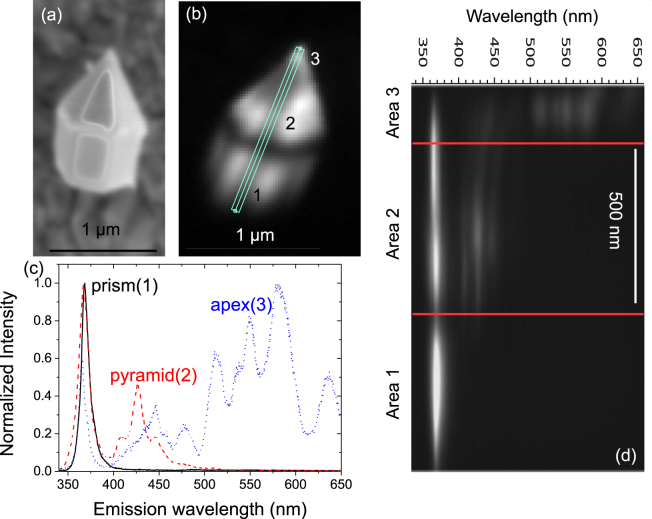
SE image (saturated due to the current needed for CL) of the nanorod (**a**), panchromatic CL intensity (**b**) and spectra (**c**) from selected spots on the nanorod as well as line spectra (**d**), taken along the line shown on the panchromatic image.

CL and scanning transmission electron microscopy (STEM) investigation of the ultra dense array revealed that the growth conditions resulted in two different, spatially separated active zones showing emission from 395 to 480 nm from the semipolar sidewalls and between 490 and 650 nm from *c*-plane QWs in the apex of the nanorods^10^. To investigate the optical properties of single nanorods and further characterize the behaviour of the different active zones, a number of nanorods were detached from the array and deposited on a copper grid, similar to previous work of our group^11^. The results of the secondary electron (SE) and CL hyperspectral imaging of a single rod are shown in Fig. 1.

The structure of the nanorod can be clearly seen in the SE image. It consists of a base with $\{10\bar{1}0\}$ (*m*-plane) sidewalls and a pyramidal top with semipolar $\{10\bar{1}1\}$ facets. The CL intensity map in Fig. 1b shows a reduction in the luminescence intensity at the point where the top (pyramid) and bottom (prism) of the nanorod meet. The intensity variations at the pyramidal top of the nanorod are largely due to re-absorption effects and the geometry of the measurement setup. Extracting the spectral information along the line shown in Fig. 1b generates a spectral line scan along the length of the nanorod. The spectral line scan is shown in Fig. 1d and three distinct regions with different optical properties can be identified.

**Figure 2:**
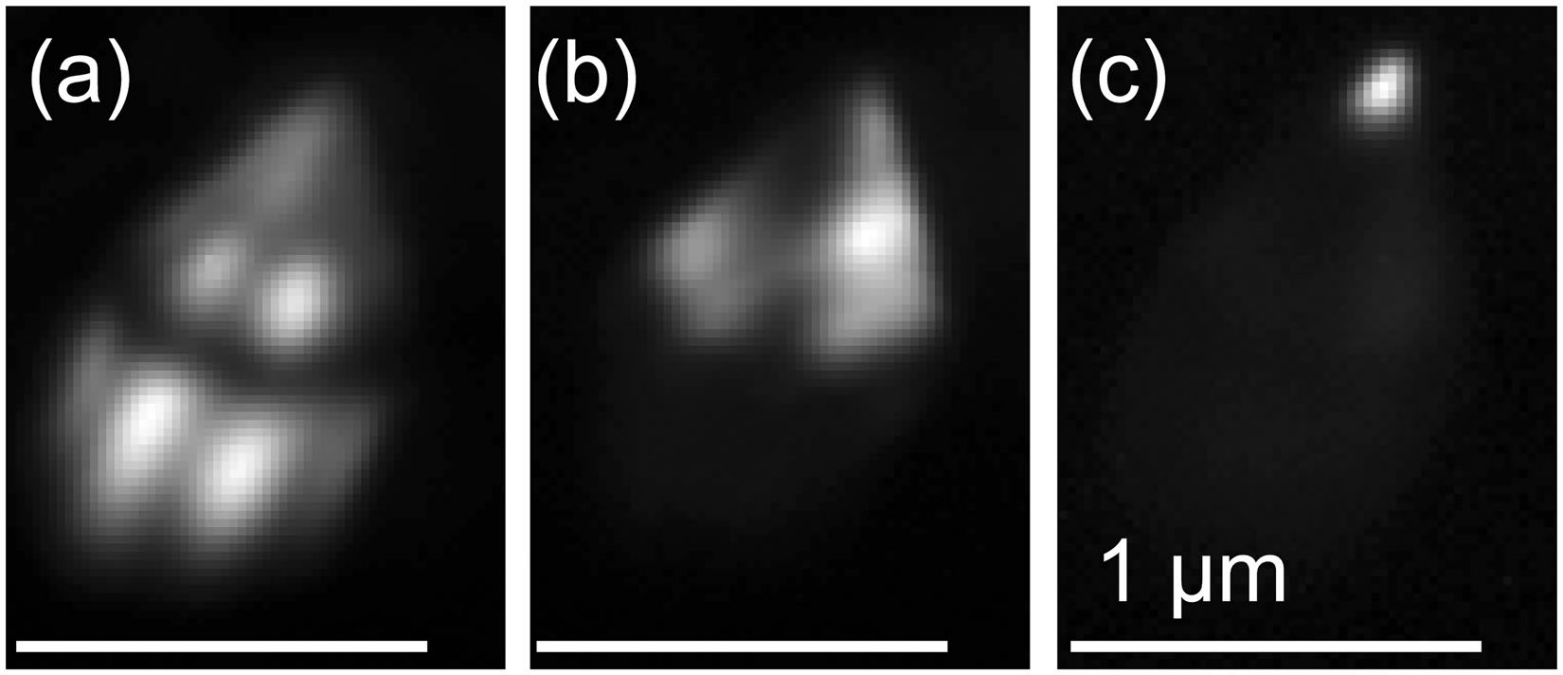
Intensity plots of the emission GaN NBE emission (**a**), the semipolar QW emission between 395 nm and 480 nm (**b**), and the emission observed from the apex of the nanorod from 490 nm to 650 nm (**c**). The scalebar is 1 μm in each case.

The first region (Fig. 1c prism(1)) is in the base of the nanorod, where only the GaN near band edge (NBE) signal can be detected, showing that no In_*x*_Ga_1−*x*_N growth occurred on the *m*-plane sidewalls during the deposition of the InGaN/GaN active zone. This is expected given the ultradense nature of the array^10^ from which the individual rod was extracted and in contrast to nanorods with a wider spacing where In_*x*_Ga_1−*x*_N growth could be found on the *m*-plane^12,13^. The small FWHM (113 meV at 300 K) of the GaN NBE peak and the absence of any yellow luminescence on the *m*-plane sidewalls indicate that damage introduced during the etching process was successfully healed during the regrowth and the material is of high optical quality^14–17^. The GaN NBE emission intensity shows some variation over the nanorod, with a slight increase in emission intensity at the ridges where two *m*-plane facets meet (see Fig. 2a). This is most likely caused by the extinction of *a*-plane facets during the regrowth^13,18^.

In addition to the GaN NBE emission near 370 nm, region 2 features a broad luminescence band (395–480 nm) consisting of a main peak with an emission wavelength of 426 nm and two satellite peaks on the high and low energy side of the main peak, with emission wavelengths of 409 nm and 446 nm respectively (Fig. 1c pyramid(2)). The three peaks are separated by an energy of approximately 150 meV. Applying a digital bandpass to the hyperspectral image to only show the luminescence intensity between 395 nm and 480 nm (Fig. 2b) it is possible to see that the luminescence of this region is emitted from the pyramidal top of the nanorod. We attribute the luminescence in this range to the semipolar QWs. As the growth conditions for the active zone were not varied throughout the growth process, only minimal variations are expected in each subsequent quantum well, thus the origin of the satellite peaks is believed to be due to optical modes such as Fabry-Perot or whispering gallery modes (WGM).

In the transition from the second region to the third region the luminescence that was associated with the semipolar QWs exhibits a redshift (see Fig. 1c), with two peaks emitting at 446 nm and 477 nm. The redshift is most likely induced by a thickness as well as InN content variation at the points at which the semipolar QWs meet the *c*-plane QWs^10^ as observed by Boulbar *et al*.^12^.

The third optical region exhibits a broad luminescence band consisting of multiple peaks from 490 nm to 650 nm covering the visible spectral range from green to red (Fig. 1d apex(3)). Applying a digital bandpass filter to the CL data to only show the luminescence intensity in this spectral range it is possible to see that this region is spatially confined to the tip of the nanorod, starting 150 nm below the apex as shown in Fig. 2c.

**Figure 3:**
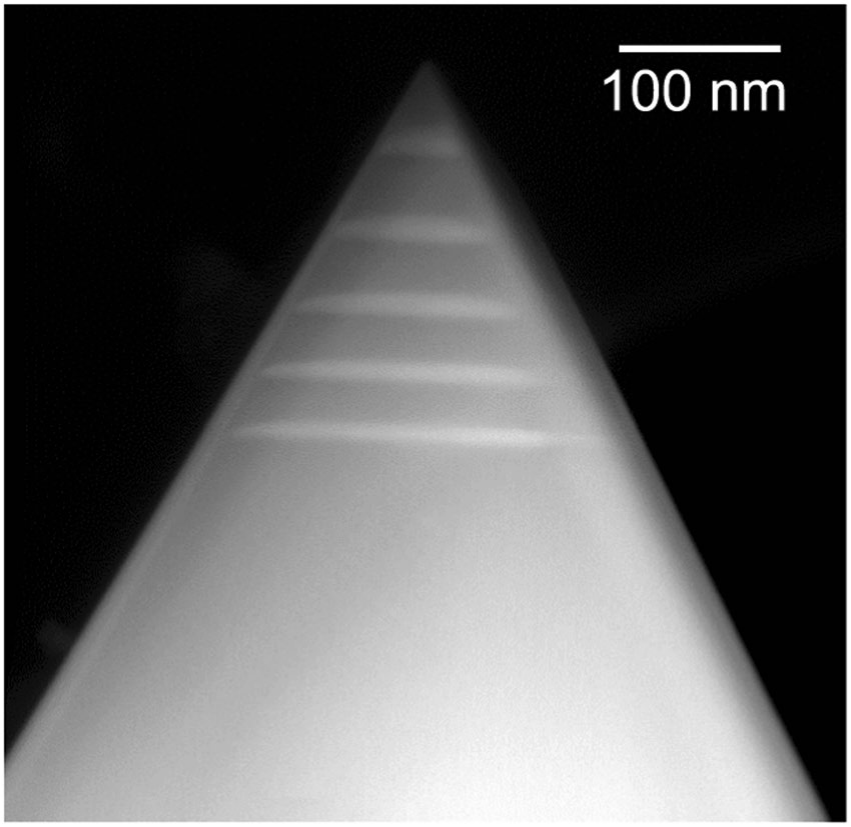
STEM image of the tip of a nanorod from the same array. Five *c*-plane QWs, with increasing thickness towards the apex, can be observed. Additionally, a decrease in QW thickness can be observed towards the pyramid edges.

STEM imaging, shown in Fig. 3, performed on a nanorod from the same array, reveals that five *c*-plane QWs have been grown at the tip of the nanorod. The growth of the *c*-plane QWs is caused by the presence of a residual *c*-plane facet on the top of the nanorod before the growth of the InGaN/GaN active zone. Emission wavelengths down to 650 nm have already been reported by Ko *et al*.^19^, who utilised a double heterostructure approach on nanopyramids, achieving a InN concentration of more than 40%. As discussed by Conroy *et al*.^10^ the InN concentration in the characterised *c*-plane QWs does not reach high enough values to account for the observed emission spectra. Instead the increase of the QW thickness to 22 nm (as seen in Fig. 3) is seen as the reason for the high wavelength emission as discussed by Liao *et al*.^20^ who attribute this red shift to a reduced quantum confinement and a stronger quantum confined Stark effect. The increase in QW thickness could explain the multiple luminescence peaks originating from the tip of the nanorod, each peak originating from a QW with different thickness (from 513 nm for the thinnest *c*-plane QW to 636 nm for the thickest QW).

However, comparing the shape of the high wavelength luminescence band with similar structures published in literature we observe smaller FWHM values (65 meV, 106 meV, 67 meV, 52 meV and 79 meV at 636 nm, 580 nm, 550 nm, 538 nm and 513 nm respectively) than the previously reported 140–130 meV at 542 nm^21^ and 400 meV at 650 nm^19^. The difference becomes even more apparent when we compare our spectrum with the results published by Kim *et al*.^22^ who observed a broad spectrum from 1.8 eV to 3.2 eV in a white light emitting core-shell nanorod. Based on this difference and the fact that the growth conditions were not optimised for *c*-plane growth we assume a broad underlying emission spectrum from the *c*-plane QWs which is modulated by optical cavity modes.

To determine which mode is causing the additional satellite peaks the energy spacing between different optical modes in the horizontal plane is calculated using Δ*E* = *hc*/*nL* with *L* the optical path length and *n* the refractive index. Assuming a constant refractive index of 2.615^23^ and a hexagonal side length *l* with a value of 350 nm, the path length *L*, of the different optical modes can be determined in order to identify which of the modes results in the energy separation closest to the one experimentally observed.

**Figure 4:**
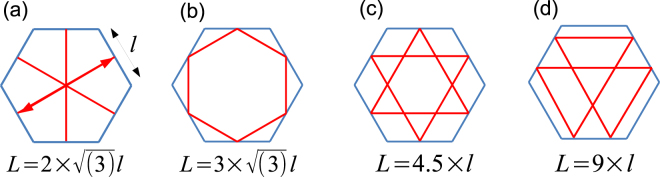
Optical modes in the horizontal plane of a hexagonal nanorod, Fabry-Perot modes (**a**), WGM (**b**), triangular quasi-WGMs (**c**), quasi-WGMs (**d**) with their corresponding optical path lengths *L*, with *l* the hexagonal side length.

Four different optical modes, which are a subset of the possible in-plane modes, have been investigated: Fabry-Perot modes (Fig. 4a), where the light strikes the sidewalls of the nanorod twice per cycle; WGM (Fig. 4b) where the light propagates around the periphery of the nanorod in a hexagonal pattern, being reflected six times; triangular quasi-WGMs (Fig. 4c) where the light is reflected three times and quasi-WGMs (Fig. 4d) where the light is reflected six times in a round trip. The estimated energy separations of the investigated modes are 350 meV, 260 meV, 306 meV and 150 meV for a, b, c and d respectively. The best agreement between the experimental energy separations and the calculated ones is found for the quasi-WGMs shown in Fig. 4d.

Using the following formula obtained from a plane wave model it is possible to calculate the peak emission wavelengths for the different quasi-WGMs^15,24^:

1 $$9l=\frac{{\lambda }_{{\rm{mode}}}}{n}\,[N+\frac{6}{\pi }\,\arctan \,(\beta \sqrt{\frac{1}{3}{n}^{2}-\frac{4}{3}})]$$

**Figure 5:**
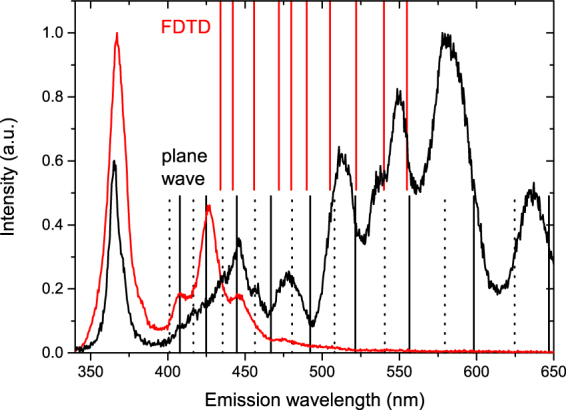
Mode positions for a nanorod with a side length of 350 nm. Results from plane wave modelling are shown as black lines, TM modes are full lines, while TE modes are dotted lines. The results of the FDTD modelling are shown as red lines. The emission from the pyramid (shown in red) and from the apex of the nanorod (shown in black) are shown for better comparison.

The left side of the equation is the physical path for the quasi-WGM, *N* is the mode number, *n* is the refractive index, which depends on the wavelength and was obtained using the values published by Adachi^23^. The last term corrects for the phase shift occurring at total internal reflection, where *β* is 1/*n* for the TM modes and *n* for TE modes^7^. The calculated values in the spectral range of the observed peaks are plotted in Fig. 5 as black lines.

The best agreement of the mode position with experimentally observed peak positions was found for a side length of 350 nm (see Fig. 5). From the calculated mode positions it can be seen that the agreement between the mode position and the experimentally observed peaks gets worse the longer the wavelength and the lower the mode number is. This is most likely due to limitations of the plane wave model or a deviation in the actual refractive index *n* from the indexes given in ref.^23^.

Due to this limitation of the plane wave model we have employed 3D FDTD modelling^25^ to investigate the mode structure (using the same values for the refractive index *n*). For this a dipole source emitting from 400 nm to 700 nm was placed into the pyramidal top of the nanorod (see Additional Information). The resulting spectrum measured by a time monitor above the nanorod is in good agreement with the plane wave model showing only small deviation from the calculated mode positions (see comparison in Fig. 5). Further investigation shows that the agreement between the FDTD and the plane wave model seems to depend on the nanorod length, a parameter that is not taken into account in the plane wave model (see Additional Information).

The good agreement between the FDTD and the plane wave model indicates that the reduction in the agreement between theory and experiment is not caused by the limitations of the plane wave model, instead we suspect a deviation of the actual refractive index of the nanorod from the refractive indexes given in ref.^23^ due to surface charges or other effects^26^.

Analysing the mode positions of the plane wave model it can be seen that for short wavelength emission TM polarized modes provide the best fit to the spectra; this changes for longer wavelength emission, where TE modes show the best agreement between the experimental data and the calculated mode positions. The good agreement with the TM polarized modes for the short wavelength emission indicates that the light in the quasi-WGMs is preferentially TM polarized. The influence of TE polarized modes on these wavelength is considerably weaker, observable in small shoulders of the three main peaks. Similar observations have been made for hexagonal and triangular ZnO and GaN cavities which have been attributed to lower losses in the TM polarized modes^5,7,27,28^. It is not clear what causes the switch of the dominant mode polarization to TE modes for longer wavelength emission, and further study of similar systems by polarization dependent CL is needed to shed light on this behaviour. A possible explanation could be the different origin of the luminescence bands coupling into the modes, although Tessarek *et al*.^29,30^ and Gong *et al*.^9^ observed both dominant TM and TE mode polarization while exciting on the *m*-plane facet of GaN nanorods and InGaN/GaN coreshell nanowires respectively.

**Figure 6:**
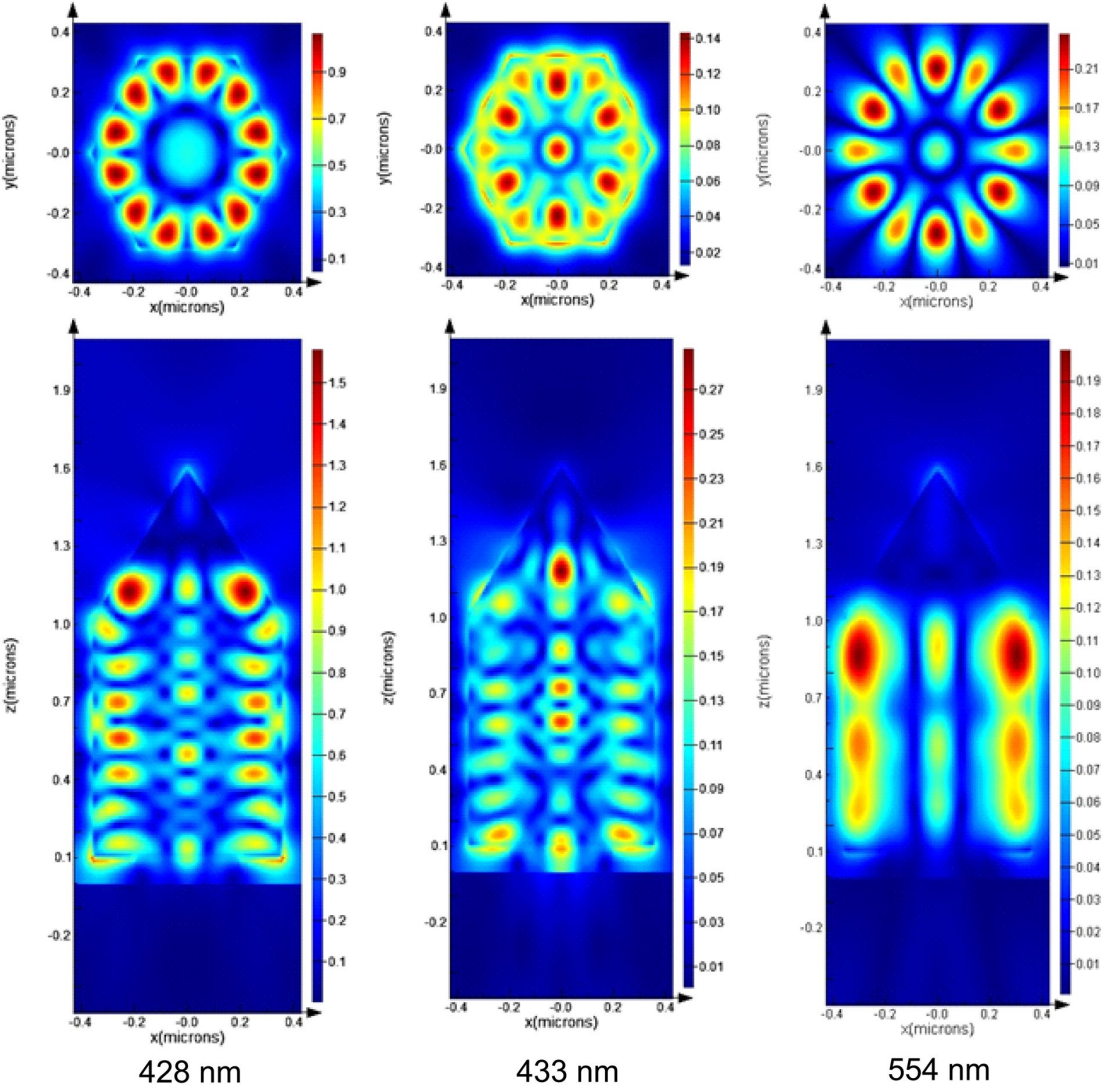
Mode E-field (magnitude) distributions for the experimentally observed resonances at around 428 nm, 433 nm and 554 nm. The source position for the first two resonances was the pyramid of the nanorod, while the source was located at the apex of the nanorod for the 554 nm resonance. The x-y plane snapshots are taken at z = 0.92 μm, and the x–z field snapshots are taken at y = 0 μm.

The main remaining question is how the light generated in the pyramidal top is modulated by the structure of the nanorod base. We attribute this to light coupling from the pyramidal top to the nanorod base which is demonstrated using 3D FDTD modelling^25^. For this a dipole source emitting from 380 nm to 480 nm was placed in the pyramidal and apex sections of the nanorod, representing the emission from the semipolar MQW region and the *c*-plane QW region respectively. The results in Fig. 6 show frequency domain snapshots of the total electric field intensity in a number of planes within the nanorod of three specific modes at 428 nm, 433 nm and 554 nm. From the FDTD simulation it can be seen that light emitted at either active region, couples into the base of the nanorod (labeled “prism” in Fig. 1).

In summary, the luminescence properties of a single In_*x*_Ga_1−*x*_N/GaN nanorod have been investigated by a combination of SE and CL hyperspectral imaging with STEM measurements and FDTD simulations. It was found that the growth resulted in the deposition of semipolar as well as polar QWs. The polar QWs are attributed to the presence of a *c*-plane facet prior to deposition of the active region, the size of which could be controlled in the GaN regrowth step^31^. FDTD simulation as well as plane wave modelling show that the QW emission is strongly influenced by optical modes propagating through the nanorod. The findings presented here show that nanorod LED structures offer an array of advantages over planar LED structures. The presence of optical modes, which can easily be engineered by changing the diameter and height of the nanorod (see Additional Information Figs. 1 and 2), as well as two QW regions emitting from the blue to the red spectral region, potentially allows the design of true white LEDs without the need of light conversion using phosphors.

## Methods

Obtaining optical information from wide band gap semiconductors on a nanometer scale is a challenging task. Photoluminescence (PL) is often used to investigate the optical properties of III-nitride semiconductors, but the spatial resolution of conventional PL is too low to investigate submicron features making it impractical for use in investigating nanostructures such as nanorods. The combination of CL and secondary electron (SE) imaging in a scanning electron microscope (SEM) enables information to be obtained on the surface morphology and the optical properties at the same time, allowing the two properties to be correlated on a nanometer scale^12,32^.

In this work we investigated a single InGaN/GaN MQW nanorod structure from an ultradense nanorod array, grown by metal organic vapor phase epitaxy (MOVPE). The sample consists of a GaN nanorod template, which was fabricated by a top-down approach, on top of which 300 nm nominally undoped GaN and a five period InGaN/GaN active region were grown. Further details on the growth of the template can be found elsewhere^10^. The active zone was grown with nominally 16% InN at 720 °C and a thickness of 2 nm. The quantum barriers consist of nominally 5 nm GaN grown at a temperature of 850 °C. The growth ultimately resulted in apex-tipped hexagonal nanorods with a hexagonal side length of 350 nm and a height of 1.5 *μ*m as determined by SE imaging^10^.

CL hyperspectral imaging^33–35^ was conducted in an environmental SEM with a 125 mm focal length spectrograph, a 600 line/mm grating and cooled charge-coupled device. The samples were tilted by 45° with respect to the incident electron beam and the generated light was collected by a reflecting objective with its optical axis perpendicular to the electron beam as described by Edwards *et al*.^36^. CL measurements were conducted, at room temperature, with an acceleration voltage of 5 kV; this results in 90% of the beam energy being deposited within a depth of 70 nm, estimated by Monte Carlo simulations using CASINO^37^.

### Data availability

The data associated with this research is available at DOI:10.15129/a24bd3fd-0493-4cd2-84ed-551abff86060

## Electronic supplementary material

Additional Information
