# Supplementary material for: Multi-wavelength emission from a single InGaN/GaN nanorod analyzed by cathodoluminescence hyperspectral imaging
Source: Sci Rep. 2018 Jan 29;8:1742. doi: 10.1038/s41598-018-20142-5 (PMC5789091; doi:10.1038/s41598-018-20142-5)
Supplement: Supplementary file 1 — Additional Information [file 41598_2018_20142_MOESM1_ESM.pdf]

Multi-wavelength emission from a single InGaN/GaN nanorod analyzed by cathodoluminescence hyperspectral imaging

Gunnar Kusch[1,*], Michele Conroy[2,3,4], Haoning Li[2,3], Paul R. Edwards[1], Chao Zhao[5], Boon S. Ooi[5], Jon Pugh[6], Martin J. Cryan[6], Peter J. Parbrook[2,3], and Robert W. Martin[1]

[1]Department of Physics, SUPA, University of Strathclyde, Glasgow G4 0NG, United Kingdom
[2]Tyndall National Institute, University College Cork, Lee Maltings, Dyke Parade,Cork, Ireland
[3]School of Engineering, University College Cork, College Road, Cork, Ireland
[4]Pacific Northwest National Laboratory, Richland, WA, United States of America
[5]Photonics Laboratory, King Abdullah University of Science and Technology (KAUST), Thuwal 23955-6900, Saudi Arabia
[6]Department of Electrical and Electronic Engineering, University of Bristol, Bristol BS8 1UB, United Kingdom
[*]gunnar.kusch@strath.ac.uk

ABSTRACT

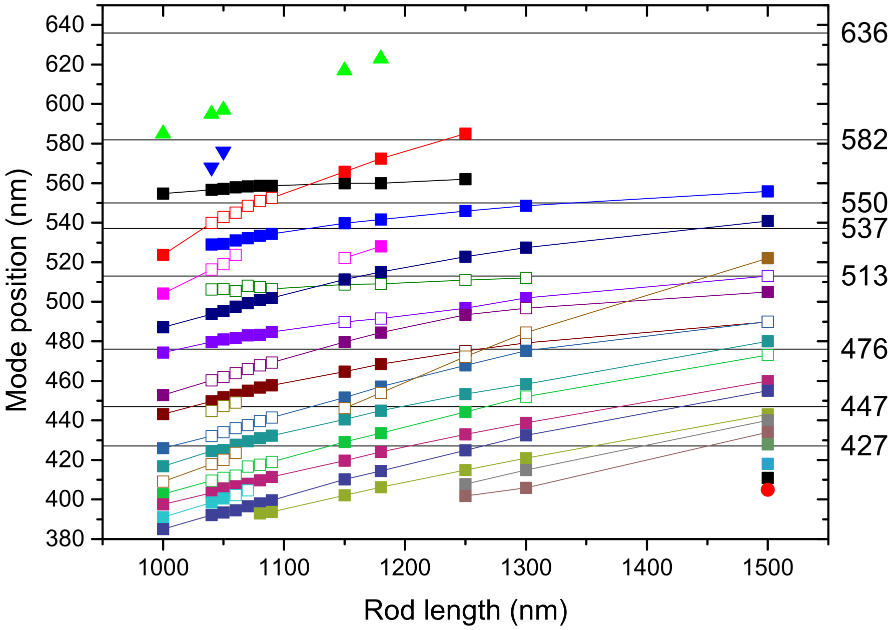

Figure 1. Mode positions as calculated by FDTD for varying nanorod length and constant nanorod diameter. Weak modes are shown as open symbols.

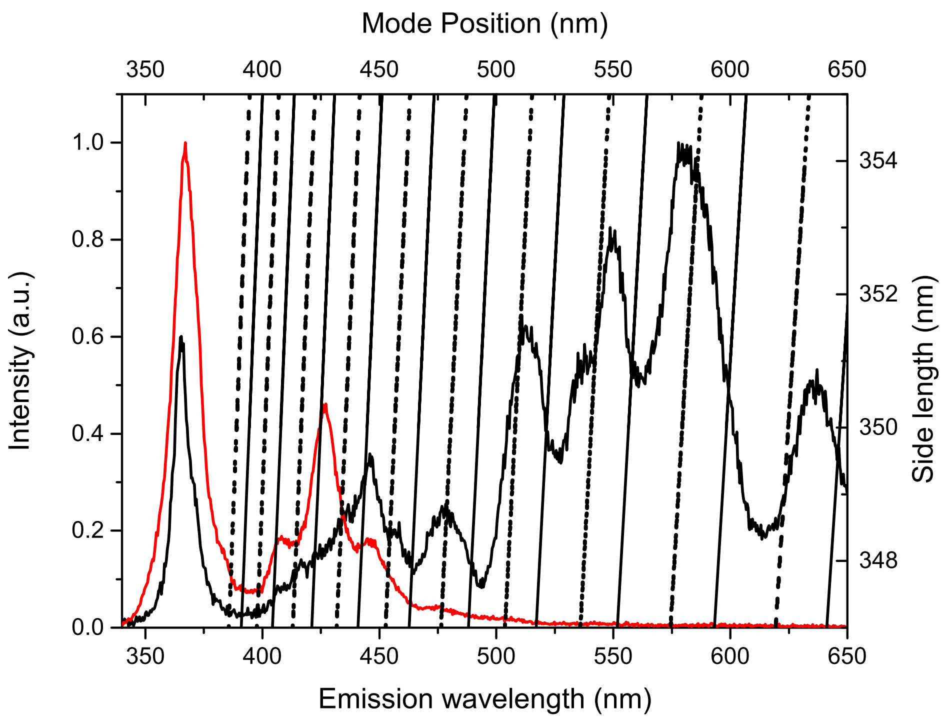

Figure 2. Mode positions as calculated by the plane wave model for different rod sidelength, TE modes are shown as dotted, TM modes as full lines.

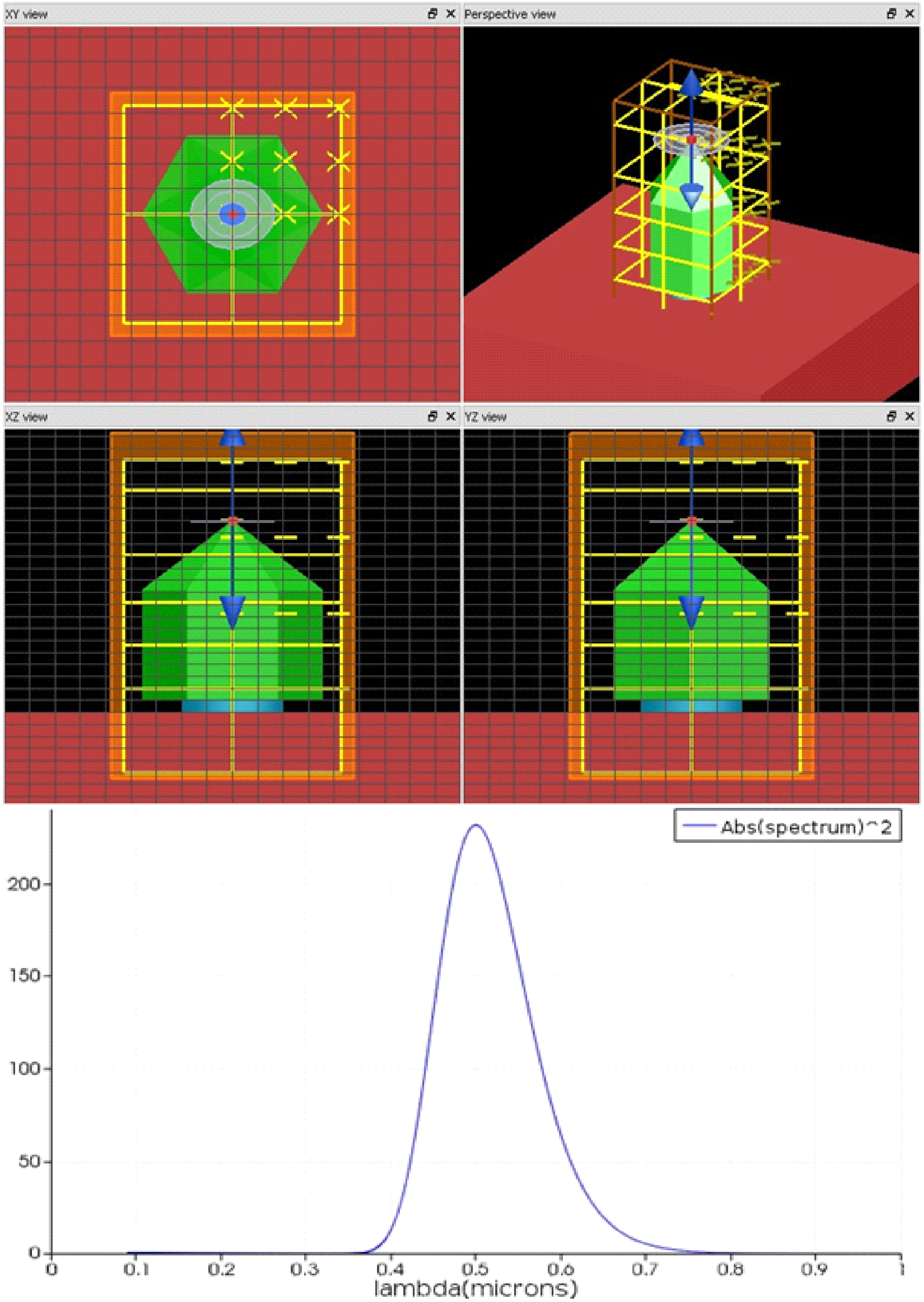

Figure 3. Nanorod geometry and input spectra used for the FDTD simulation of the nanorod. The source in the shown case is located in the apex of the nanorod representing the *c*-plane QW emission, for the modelling of the behaviour of the semipolar QWs the source was moved into the pyramidal section of the nanorod.
